# Multidrug resistance pattern of bacterial agents isolated from patient with chronic sinusitis

**Published:** 2016

**Authors:** Mohammad Sadegh Rezai, Rostam Pourmousa, Roksana Dadashzadeh, Fatemeh Ahangarkani

**Affiliations:** 1 Infection Diseases Research Center with Focus on Nosocomial Infection, Mazandaran University of Medical Sciences, Sari, Iran.; 2 Antimicrobial Resistance Research Center, Department of Infectious Diseases, Mazandaran University of Medical Sciences, Sari, Iran.

**Keywords:** Chronic sinusitis, Bacteria, Antibiotic resistance, MRSA, ESBL, MDR

## Abstract

**Background:**

Treatment of chronic sinusitis is complicated due to increase of antibiotic-resistant bacteria. The aim of this study was to determine the multidrug resistance (MDR) pattern of the bacteria causing chronic sinusitis in north of Iran.

**Methods:**

This cross-sectional study was carried out on patients with chronic sinusitis. Bacterial susceptibility to antimicrobial agents was determined according to the CLSI 2013 standards. Double-disk synergy (DDS) test was performed for the detection of extended-spectrum beta-lactamase (ESBL) producing bacteria; also methicillin-resistant Staphylococcus (MRSA) strains were identified by MRSA screen agar. The MDR isolates were defined as resistant to 3 or more antibiotics. Data were analyzed using SPSS 17 software. Descriptive statistics was used to describe the features of the data in this study.

**Results:**

The rate of ESBL-producing bacteria was 28.75-37.03% among enterobacteriaceae and the rate of MRSA was 42.75%-60% among *Staphylococcus* strains. The most detectable rate of the MDR bacterial isolates was Gram-negative bacteria 39 (76.47%) and *Enterobacter *spp. 19(70.37%) was the most multidrug resistant isolate among Gram negative bacteria. Also 36 (73.46%) of the gram positive bacterial isolated were multidrug resistance and *Staphylococcus aureus* 9(90%) was the most MDR among Gram positive bacteria.

**Conclusion:**

Antimicrobial resistance is increasing in chronic bacterial sinusitis. The emergence of MRSA and ESBL bacteria causing chronic sinusitis is increasing.

Treatment of chronic sinusitis, as one of the more prevalent chronic illnesses is complicated due to increase of antibiotic-resistant bacteria (1, 2). Recent reports have shown the emergence of antibiotic resistance bacteria in sinusitis and the increasing rate of multidrug resistance (MDR) enterobacteriaceae causing chronic sinusitis (3-5). Extended-spectrum beta-lactamases (ESBLs) and methicillin-resistant Staphylococcus strains (MRSA) represent a major threat among MDR bacterial isolates (6). There are limited reports regarding the prevalence of MRSA in the setting of chronic sinusitis (7). Treatment of chronic sinus infection associated with the recovery of MRSA is challenging. It is important to provide coverage against these organisms as well as against other potential aerobic and anaerobic bacteria (8-10) anaerobic bacteria and *Staphylococcus aureus* are more considerable bacteria in chronic sinusitis. Penicillin group, amoxicillin or amoxicillin-clavulanate are effective against bacteria causing sinusitis. There is increasing rate of MDR bacteria among respiratory pathogens, including pneumococci and *H. influenzae*. Resistance rates vary regionally (11, 12).

Empiric antibiotic therapy is often used for sinusitis, on the other hand, the emergence of antibiotic resistance has increased the failure rate of this approach (13). The epidemiology of bacteria causing chronic sinusitis in European and American countries differs from Iran and in these countries people routinely vaccinate* Hemophilus influenzae* and pneumococcus. In our knowledge, not only there are limited data on common pathogens associated with sinusitis in Iranian patients, but also no comprehensive information exists on antimicrobial susceptibility to common antibiotics used for treating sinusitis. (14-16). In view of the increased resistance to antibiotics in several upper airway bacteria, the aim of this study was to determine the multidrug resistance(MDR) pattern of the bacteria causing chronic sinusitis in north of Iran**.**

## Methods

This cross-sectional study was carried out on 100 patients with chronic sinusitis. The endoscopy method was used for bacterial sampling. The standard microbiological procedures were performed for the identification of the bacteria causing chronic sinusitis (17, 18). Bacterial susceptibility to antimicrobial agents was determined by the Kirby–Bauer method according to the CLSI 2013 standards. Inoculums were diluted to final concentration (5*10^5^ CFU/ ml) and inoculated into Mueller-Hinton agar (19). ESBL-producing enterobacteriaceae was detected using the double-disk synergy (DDS) test. ESBL presence was assayed using the following antibiotic disks (MAST, UK): ceftazidime/ clavulanic acid (30/10 μg) cefotaxime (30 μg), cefotaxime/ clavulanic acid (30/10 μg) and ceftazidime (30 μg). *Escherichia coli* ATCC 25922 strain served as positive control. 

Oxacillin screen agar was used for detection of methicillin-resistant Staphylococcus strains. Staphylococcus strains (MRSA) were cultured on Muller Hinton agar containing 4% NaCl and 6 mg/L oxacillin and were incubated for 18-24 hours (20, 21). The tested antibiotics were penicillin (P), amoxicillin (AMX), ampicillin (AM), vancomycin (V), oxaciline (OXA), gentamicin (GM), cefuroxime  (CXM), cefazolin (CZ), ceftriaxone (CRO), ceftizoxime (CT), ciprofloxacin (CP), co-trimoxazole (SXT), amikacin (AN). MDR isolates were defined resistant to three or more antibiotics (22). Data were analyzed using SPSS 17 software. Descriptive statistics was used to ascribe the data of the study.

## Results

Out of total number of patients, 100 subjects were evaluated in our study .58 were males (58%) and 42 were females (42%) (P=.78). The average age was 34.2±11.1 (range 8 year, 68 year). The prevalence of ESBL bacteria for *Enterobacter *spp., *Escherichia* *coli* and *Klebsiella Pneumonia *were 10 (37.3%), 5 (29.41%) and 2 (28.57), respectively. The rates of MRSA Staphylococcus strains for *S.aureus* and *S. epidermidis* were 6 (60%) and 14 (43.75%). Antibiotic susceptibility pattern of bacterial agents is shown in table 1. 

**Table 1:** Antibiotic susceptibility pattern of bacterial agents

Oxaciline	Ciprofloxacin	Ceftizoxime	Ceftriaxone	Cefazolin	cefuroxime	Corimonazal	Amikacin	Gentamicin	vancomycin	Ampicillin	Amoxicillin	penicillin		Antibiotics Bacteria	
40.62	25.8	16.12	25.8	9.67	32.25	-	-	9.67	6.45	25.8	32.25	16.12	R		*Staphylococcus* *epidermidis *N=32
18.75	29.03	32.25	29.03	38.7	6.67	-	-	38.7	41.93	12.9	6.67	12.9	S		
60	30	10	40	30	20	-	-	50	20	60	60	40	R		*Staphylococcus* *aureus* N=10
20	40	60	30	30	40	-	-	20	40	10	10	30	S		
100	57.1	57.1	57.1	57.1	57.1	-	-	-	57.1	42.8	-	42.8	R		*Streptococcus* beta hemolytic group A N=7
0	-	-	-	-	-	-	-	-	-	-	-	14.28	S		
-	28.6	14.3	28.6	14.3	-28.6	14.3	14.3	28.6	57.1	-	42.8	-	R		*Klebsiella* *pneumoniae*N=7
-	28.6	42.85	28.57	57.14	-	42.85	57.14	42.85	-	-	14.3	-	S		
-	29.4	23.5	23.5	29.4	41.17	35.3	17.6	-	41.17	0	0	0	R		*Escherichia* *coli *N=17
-	17.64	23.52	23.52	17.64	-	11.76	29.41	47.05	5.9	-	-	-	S		
-	37	33.3	33.3	48.1	29.62	33.3	37	18.5	-	-	-	-	R		*Enterobacter* spp. N=27
-	11.11	14.81	14.81	0	33.3	14.81	11.11	29.62	-	-	-	-	S		

R=resistance S=sensitive

Multi drug resistance pattern of bacterial agents is shown in table 2. The most frequently isolated organisms from sinus and nasopharyngeal were; Gram-positive bacilli 70 (40 sinus and 30 nasopharyngeal) *Staphylococcus epidermidis* 32 (17 sinus and 15 nasopharyngeal), *Enterobacter *spp. 27 (13 sinus and 14 nasopharyngeal), *Escherichia coli* 17 (8 sinus and 9 nasopharyngeal),* Staphylococcus aureus *10 (7 sinus&3 nasopharyngeal), streptococcus beta hemolytic group A 7 (4 sinus and 3 nasopharyngeal) and *Klebsiella pneumonia* 7 (3 sinus and 4 nasopharyngeal) *Candida albicans* 1 (0 sinus &1 nasopharyngeal) and11 samples were negative culture (3 sinus and 8 nasopharyngeal).

**Table 2.** Multi-drug resistance pattern of bacterial agents

**Bacteria**	**Antibiotics** **No**	**Antibiotic resistant isolates** **No (%)**	**Antibiotics**
*Staphylococcus* *epidermidis* N=32	11	2 (6.25)	P, AMX, AM, V, OXA, GM, CXM , CZ, CRO, CT, CP
	10	3 (9.37)	P, AMX, AM, OXA, GM, CXM , CZ, CRO, CT, CP
	8	4 (12.5)	P, AMX, AM, OXA, CXM , CRO, CT, CP
	7	4 (12.5)	P, AMX, AM, OXA, CRO, CT, CP
	4	6 (18.75)	AMX, AM, CRO, CP
	3	3 (9.37)	AMX ,CRO, CP
*Staphylococcus* *aureus *N=10	11	1 (10)	P, AMX, AM, V, OXA, GM, CXM , CZ, CRO, CT, CP
	10	2 (20)	P, AMX, AM, V, OXA, GM, CXM , CZ, CRO, CP
	8	2 (20)	P, AMX, AM, OXA, GM, CZ, CRO, CP
	6	2 (20)	P, AMX, AM, OXA, GM, CRO
	4	1 (10)	P,AMX, AM, GM
	3	1 (10)	P, AMX, AM
*streptococcus* beta hemolytic group A N=7	8	3 (42.85)	P, OXA, GM, CXM , CZ, CRO, CT, CP
	7	3 (42.85)	OXA, GM, CXM , CZ, CRO, CT, CP
*Klebsiella* *Pneumonia *N=7	7	3 (57.14)	CP, CRO, CZ, CXM , SXT, AN, GM
	3	4 (71.42)	CP, CRO, GM
*Escherichia* *Coli *N=17	6	6 (35.29)	AN, SXT, CZ, CRO, CT, CP
	5	5 (29.41)	SXT, CZ, CRO, CT, CP
	3	2 (11.76)	SXT, CZ, CP
*Enterobacter* spp. N=27	7	7 (25.92)	GM, AN, SXT, CZ, CRO, CT, CP
	6	6 (22.22)	AN, SXT, CZ, CRO, CT, CP
	3	6 (22.22)	AN, CZ, CP

## Discussion

Our results showed the increased role of MRSA and ESBL-producing bacteria in chronic sinusitis. Total of 20(47.61%) of Staphylococcus strains were MRSA and 17(33.33%) of enterobacteriaceae isolates were ESBL-producing bacteria. Increasing MRSA strains is a major problem in the hospital and community settings (23). We found that *S. aureus* has very high rate of antibiotic resistance. For example we reported 60% MRSA and 20% vancomycin-resistant *S. aureus*. Manarey identified 9.22% incidence of MRSA-causing chronic sinusitis (24). Brook et al. compared the rate of recovery of methicillin-resistant *S. aureus *between the periods 2001-2003 and 2004–2006. *S. aureus* was found in 15% of the patients with chronic sinusitis between 2001 and 2003, 27% were MRSA, and from 20% of the patients with chronic sinusitis during 2004-2006, 61% were MRSA (*P*<0.05) (9). Similar to our findings, Davoudi et al. reported resistance to vancomycin observed in one isolated *S. aureus* and the rate of MRSA* S. aureus* was 54.2% (21). Motamedi et al. and Dibah et al. reported the incidence rate of MRSA *S. aureus* of 25% and 46%, respectively (25, 26). All isolates were susceptible to vancomycin in Dibah et al.’s research (26). Overall, the data illustrated that a significant increase occurred in the rate of MRSA in patients with chronic sinusitis. Outpatient intravenous antibiotics may be an effective therapy for the treatment of MRSA sinusitis (27). Community-acquired MRSA sinusitis can be treated on an outpatient basis with culture-directed oral antibiotics (28).

The rate of ESBL-producing bacteria was 28.75- 37.03% among enterobacteriaceae in our study. Studies on patients infected by ESBL producing enterobacteriaceae have been shown to share several common factors. The presence of these risk factors should alert the attending physician to the possibility of an ESBL-related infection: Device related (arterial catheters; central venous catheters; urinary tract catheters; gastrostomy or jejunostomy tube; umbilical catheters), surgical related, antibiotic exposure (3rd generation cephalosporins (especially ceftazidime); fluoroquinolones; trimetroprim- sulfamethoxazole), previous nursing home residence, prolonged duration of hospital or ICU longer stay is associated with more severe underlying diseases, with invasive procedures and with antibiotic administration and severity of illness (22, 29-32). Infections with ESBL-producing organisms are usually hospital-acquired. In our study, none of the patients had nosocomial infections and it seems only probable risk factor for the spread of ESBL bacteria was indiscriminate use of antibiotics such as amoxicillin,** c**efixime, azithromycin, ciprofloxacin, cotrimoxazole and so on. Many surveillance studies performed in Europe and other geographical regions showed an increased prevalence and dispersion of ESBL producing isolates (6). Similar to our findings, Rezai et al. reported 30.5% ESBL-producing *E.coli *(33)*. *On one hand Akyar in Turkey found 12% ESBL-producing E.coli and *Klebsiella pneumonia *(34)*. *Also, Heffernan in New Zealand, estimated species distribution among the ESBL- enterobacteriaceae in 2012, the survey reported: 61.7% *E. coli*, 33.5% Klebsiella species and 2.8% Enterobacter species (35). Carbapenems such as imipenem or meropenem have been shown to be associated with the lowest mortality of any drug class when used against infections with ESBL-producing organisms (36).

The most detectable rate of MDR bacteria isolated were Gram negative bacteria 39(76.47%) and *Enterobacter *spp. was the most MDR isolates, also the antibiotic susceptibility test showed that 36 (73.46%) of the Gram positive isolates were MDR and *Staphylococcus aureus* 9(90%) was the most multidrug resistant among Gram positive bacteria. We found high prevalence of MDR bacteria among enterobacteriaceae family in our study, especially all *Klebsiella Pneumonia *isolated were MDR. In our study, the rate of MDR for *E.coli *(76.47%) and *Enterobacter *spp. (%70.37) was high in comparison to Rezai et al. and Pradel et al.’s findings (33, 37). We reported the sensitivity to antibiotics only in 9% of cases to 8-10 antibiotics and the total number of 76% microbial species was resistant to at least three antibiotic discs. In our study, we reported that resistance to cephalosporins such as cefuroxime, cefazolin, ceftazidime and ceftriaxone among enterobacteriaceae family was in average level similar to other studies (30, 38-40). Antibiotic choices in the treatment of chronic sinusitis should be guided by sinus cultures whenever possible, and the use of broader single antibiotic agent should be considered with or without the addition of anaerobic coverage antibiotics (41). Antimicrobial resistance is increasing in bacteria causing chronic sinusitis especially to beta-lactam antibiotics among Gram positive bacteria in our study. The emergence of MRSA and ESBL bacteria causing chronic sinusitis is increasing. In addition, this resistance affects the efficiency of different generations of cephalosporins in the treatment of chronic sinusitis.

## Notes

**Funding:** This article is result of Dr. Roksana Dadashzadeh’s thesis with 90-9 grant number.
